# TISU: Extracorporeal shockwave lithotripsy, as first treatment option, compared with direct progression to ureteroscopic treatment, for ureteric stones: study protocol for a randomised controlled trial

**DOI:** 10.1186/s13063-018-2652-1

**Published:** 2018-05-22

**Authors:** Samuel McClinton, Sarah Cameron, Kathryn Starr, Ruth Thomas, Graeme MacLennan, Alison McDonald, Thomas Lam, James N’Dow, Mary Kilonzo, Robert Pickard, Ken Anson, Frank Keeley, Neil Burgess, Charles Terry Clark, Sara MacLennan, John Norrie, for the TISU Study Group

**Affiliations:** 1 Aberdeen Royal Infirmary, Foresterhill, Aberdeen, AB25 2ZN UK; 2 Academic Urology Unit, University of Aberdeen, Health Sciences Building, Foresterhill, Aberdeen, AB25 2ZD UK; 3 The Centre for Healthcare Randomised Trials (CHaRT), Health Services Research Unit, University of Aberdeen, Health Sciences Building, Foresterhill, Aberdeen, AB25 2ZD UK; 4 Health Economics Research Unit, University of Aberdeen, Polwarth Building, Foresterhill, Aberdeen, AB25 2ZD UK; 5 Institute of Cellular Medicine, Newcastle University, Newcastle Upon Tyne, NE2 4HH UK; 6 St George’s Hospital, London, SW17 0QT UK; 7 Southmead Hospital, Bristol, BS10 5NB UK; 8 Norfolk and Norwich University Hospitals, Colney Lane, Norwich, NR4 7UY UK; 9 Stone Patient Advisory Group, Section of Endourology, British Association of Urological Surgeons, London, UK; 10 Edinburgh Trial Unit, University of Edinburgh, Edinburgh, EH16 4UX UK

**Keywords:** Ureteric stone, ESWL, Ureteroscopy

## Abstract

**Background:**

Urinary stone disease is very common with an estimated prevalence among the general population of 2–3%. Ureteric stones are associated with severe pain as they pass through the urinary tract and have significant impact on patients’ quality of life due to the detrimental effect on their ability to work and need for hospitalisation. Most ureteric stones can be expected to pass spontaneously with supportive care. However, between one-fifth and one-third of cases require an intervention.

The two standard active intervention options are extracorporeal shockwave lithotripsy (ESWL) and ureteroscopic stone retrieval. ESWL and ureteroscopy are effective in terms of stone clearance; however, they differ in terms of invasiveness, anaesthetic requirement, treatment setting, complications, patient-reported outcomes (e.g. pain after intervention, time off work) and cost. There is uncertainty around which is the most clinically effective in terms of stone clearance and the true cost to the NHS and to society (in terms of impact on patient-reported health and economic burden).

The aim of this trial is to determine whether, in adults with ureteric stones, judged to require active intervention, ESWL is not inferior and is more cost-effective compared to ureteroscopic treatment as the initial management option.

**Methods:**

The TISU study is a pragmatic multicentre non-inferiority randomised controlled trial of ESWL as the first treatment option compared with direct progression to ureteroscopic treatment for ureteric stones.

Patients aged over 16 years with a ureteric stone confirmed by non-contrast computed tomography of the kidney, ureter and bladder (CTKUB) will be randomised to either ESWL or ureteroscopy. The primary clinical outcome is resolution of the stone episode (no further intervention required to facilitate stone clearance) up to six months from randomisation. The primary economic outcome is the incremental cost per quality-adjusted life years (QALYs) gained at six months from randomisation.

**Discussion:**

Determining whether ESWL is not inferior clinically and is cost-effective compared to ureteroscopic treatment as the initial management in adults with ureteric stones who are judged to require active treatment is relevant not only to patients and clinicians but also to healthcare providers, both in the UK and globally.

**Trial registration:**

ISRCTN registry, ISRCTN92289221. Registered on 21 February 2013.

**Electronic supplementary material:**

The online version of this article (10.1186/s13063-018-2652-1) contains supplementary material, which is available to authorized users.

## Background

Urinary stone disease is very common with an estimated prevalence among the general population of 2–3% (1.8 million people in the UK) with males forming stones three times as often as females [1]. Urinary stones often recur and the lifetime recurrence rate is approximately 50% [2]. The interval between recurrences is variable, with approximately 10% within one year, 35% within five years and 50% within ten years [3]. The increased incidence of urinary stones in the industrialised world is associated with improved standards of living (mainly due to the high dietary intake of proteins and minerals) and there is also an association with ethnicity and region of residence [4]. Urinary tract stones, and ureteric stones in particular, are associated with severe pain as they pass through the urinary tract and can have a significant impact on patients’ quality of life due to the detrimental effect on their ability to work and the need for hospitalisation.

Urinary stones are a major burden on the NHS resulting in over 84,323 finished consultant episodes and over 97,558 bed-days in England in 2011–2012 [5]. When urinary stones move from the kidney into the ureter (tube connecting the kidney to the bladder), they cause severe debilitating pain (ureteric colic) that causes a large transient impairment of quality of life and leads to substantial calls on health service resources. Ureteric colic is the most common cause of emergency admission to Urology departments in the UK [5] and since it predominantly affects younger people (aged 16–55 years) is a common cause of time off work. The aim of treatment for ureteric stones is the immediate relief of symptoms, decompression of the urinary tract and the achievement of clinically complete stone clearance.

Most ureteric stones can be expected to pass spontaneously with supportive care (painkillers and fluids), so-called conservative management. Between one-fifth and one-third of cases require an active intervention (stone removal) because of failure to pass the stone, continuing pain, infection or obstruction to urine drainage. The two standard active intervention options are extracorporeal shockwave lithotripsy (ESWL) and ureteroscopic stone retrieval. While both ESWL and ureteroscopy appear to be effective in terms of stone clearance they differ in terms of invasiveness, anaesthetic requirement, treatment setting, the number of procedures required to clear the stone, complications, patient-reported outcomes (such as severity and duration of pain after intervention, time off work and bothersome urinary symptoms) and cost. There is uncertainty around which is the most clinically effective in terms of stone clearance and the true cost to the NHS and to society (in terms of impact on patient-reported health and economic burden).

A joint clinical guideline on the management of ureteric stones by the European Association of Urology and the American Urological Association [6] estimates that 68% of stones ≤ 5 mm and 47% of stones 5–10 mm in size can be expected to pass spontaneously and concluded that the majority of these stones pass within four to six weeks of presentation. Stones in the distal ureter pass more readily than stones located more proximally. Consequently, patients with favourable features and with smaller sized stones in the lower ureter are initially treated conservatively. Immediate active intervention occurs in those patients with larger stones and unfavourable features who are deemed clinically to be unsuitable for conservative treatment. Those who fail standard conservative care or who subsequently develop complications also undergo later active treatment. This can be ESWL, preliminary ureteric stenting with later stone removal, ureteroscopy with stone retrieval or destruction (in situ lithotripsy) or percutaneous nephrostomy insertion and later stone removal. ESWL and ureteroscopic treatment require expensive equipment and urological expertise. Both have been shown to be options that are safe and effective in a number of studies. In clinical practice, urologists tend to favour ureteroscopy over ESWL particularly for mid and lower ureteric stones due to perceived higher rate of clinical stone clearance [7].

A Cochrane systematic review (2007) [8] comparing the effectiveness of ESWL with ureteroscopic management of ureteric stones identified seven randomised controlled trials (RCTs) involving a total of 732 participants. The results of this review suggest stone-free rates were lower in the ESWL group (RR (relative risk) = 0.83, 95% confidence interval [CI] = 0.70–0.98); reflecting this retreatment, rates for ESWL were higher (RR = 2.78, 95% CI = 0.53–14.71) but these findings are associated with much uncertainty. Complications were less frequent after ESWL (RR = 0.44, 95% CI = 0.21–0.92) and this option was associated with a shorter hospital stay (mean difference = 2.10 days, 95% CI = – 2.55 – – 1.64 days). The review concluded that ureteroscopic treatment of stones was associated with a higher stone free rate but a higher morbidity (complication rate) and a longer hospital stay. However, the overall quality of the studies was poor and inclusion criteria were strict in each study, limiting both the generalisability and applicability of the review findings. There was limited evidence on which to judge the comparative effectiveness in clinically important prognostic subgroups for example location of stones in the ureter. None of the studies reported on health-related quality of life and only one reported a cost-effectiveness outcome. The review authors recommended that a large-scale multicentre RCT was needed to adequately address the effectiveness and cost-effectiveness of ureteroscopy versus ESWL. A 2011 update of the Cochrane review [9] has included two further studies but these have not altered the previous conclusions.

The European Association of Urology and the American Urological Association (EAU-AUA) Clinical Guideline Panel on the management of ureteric stones also conducted a review and meta-analysis reporting clinical outcomes and complications following treatment of ureteric stones with ESWL or ureteroscopy, including data from non-randomised comparisons and case series [6]. Pre-defined outcome measures were stone-free rate and number of additional procedures required. The results were stratified according to stone location in the ureter (proximal, mid, distal) and stone diameter (≤ 10 mm, > 10 mm). All forms of ESWL were analysed as a single treatment modality in the meta-analysis. The Panel concluded that the main advantage of ureteroscopy is a higher stone-free rate with a single procedure, but with a higher complication rate. However, the evidence was insufficient for the Panel to recommend between ESWL and ureteroscopy and concluded that for patients requiring active stone removal, either treatment modality is acceptable as first-line options. For the individual patient, the choice is often determined by a number of factors including the availability of resources, preference of the treating urologist, and preference of the patient. The review highlighted design and reporting deficiencies from available studies, including poor definition of stone size, inconsistent reporting of outcomes and lack of randomisation. One of the main recommendations of the Panel was the need to conduct RCTs comparing the clinical and cost-effectiveness of ESWL and ureteroscopy.

In response to the research questions raised by these evidence summaries, the TISU trial will provide the high-quality evidence, from a large pragmatic RCT, on the relative effectiveness, and cost-effectiveness, of ESWL and ureteroscopy, that will inform patients, clinicians and policy makers on the optimal choice of intervention for ureteric stones.

The aim of this trial is to determine whether in adults with ureteric stones judged to require active treatment, ESWL is not inferior, clinically, and is  more cost-effective compared to ureteroscopic treatment as the initial management option.

The hypothesis being tested is that the outcome in patients receiving ESWL as their first treatment option is not inferior to outcome in patients receiving direct ureteroscopic retrieval. The clinical and cost-effectiveness will be determined with respect to:

i. Resolution of stone episode (stone clearance), defined as no further intervention required to facilitate stone passage;
ii. Incremental cost per quality-adjusted life years (QALYs);
iii. Participant-reported health outcomes; and
iv. Disease or treatment-related harms up to six months post randomisation.

## Methods and design

**Fig. 1:**
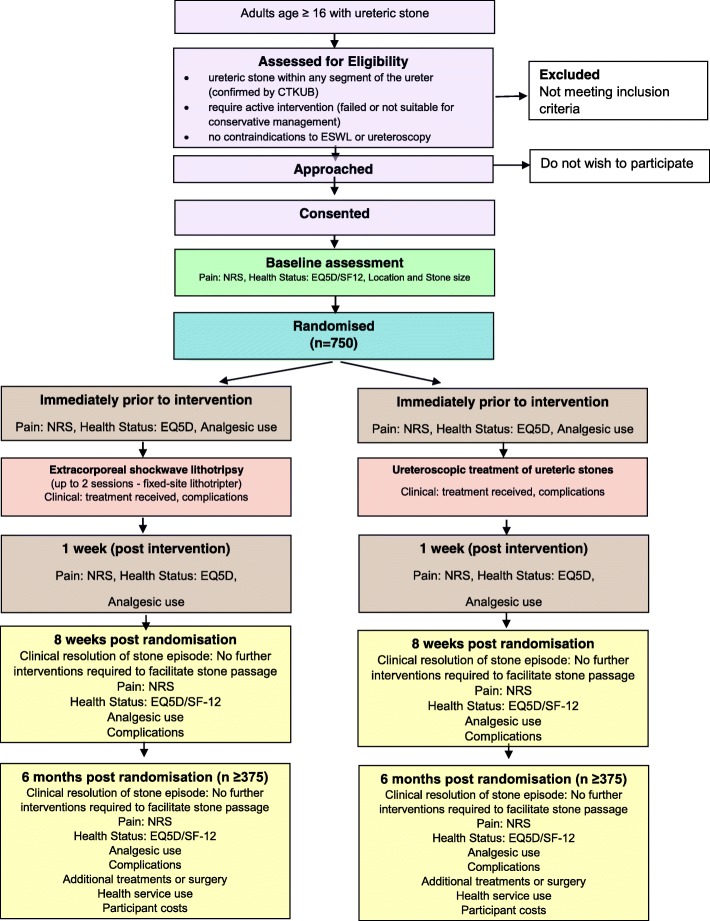
*Flow diagram*. Randomised controlled trial comparing extracorporeal shockwave lithotripsy (ESWL) with ureteroscopic retrieval as first treatment options for urinary stones

A pragmatic, multicentre, non-inferiority RCT of ESWL as the first treatment option compared with direct progression to ureteroscopic treatment for ureteric stones. The trial will be conducted in secondary care units with a high volume of ureteric stones across the UK and at sites that have a fixed lithotripter. Figure 1 summarises the trial design.

### Inclusion and exclusion criteria

#### Inclusion criteria

- Presence of stone confirmed by CTKUB
- Patients with a ureteric stone requiring removal
- Adults aged ≥ 16 years
- Single ureteric stone requiring treatment
- Suitable for either ESWL or ureteroscopic treatment
- Capable of giving written informed consent, which includes adherence with the requirements of the trial

#### Exclusion criteria

- Pregnancy
- Stones not confirmed by CTKUB
- Bilateral ureteric stones
- Patients with abnormal urinary tract anatomy (such as horseshoe kidney or ileal conduit)
- Patients unable to understand or complete trial documentation

### Trial interventions

Two interventions will be evaluated: (1) ESWL and (2) ureteroscopy.

### Identification and enrolment of potential participants

Local procedures at participating hospitals are different; the timing and mode of approach to patients and the consent process will vary to accommodate both the variability at the sites and needs of the patient. As standard practice, clinicians or delegated personnel will assess patients presenting with suspected ureteric calculi. A log will be taken of all patients assessed in order to document the reasons for non-inclusion in the study (e.g. reason they were ineligible or declined to participate) to inform the CONSORT diagram. Brief details of potentially eligible patients will be recorded in the screening logs at each site (these will be an aid to monitoring potential participant inclusion). Following adequate pain relief and confirmation of ureteric calculi by CTKUB, eligible patients (according to the inclusion and exclusion criteria) will be provided a patient information leaflet. The information leaflet will be given to each potential participant to inform them of the benefits and known drawbacks of all aspects of this trial. The PIL explains that the trial is investigating the use of either ESWL or ureteroscopic treatment as the first treatment option for symptomatic ureteric stones that require an active intervention. Each patient will have the opportunity to discuss the study with the local clinical team. Patients may make a decision to participate, during a consultation with the local clinical team during a visit to hospital (e.g. when they attend a clinic appointment or while a patient in hospital for their initial stone episode) or alternatively at home. If the patient agrees to be contacted at home, he/she may receive a telephone call from the local Research Nurse to discuss any queries. Signed informed consent forms will be obtained from the participants in all centres. Participants who cannot give informed consent (e.g. due to incapacity) will be not be eligible for participation. The participant’s permission will be sought to inform their general practitioner that they are taking part in this trial. Patients who decide to participate following telephone counselling can either send their completed documents (consent form and baseline questionnaire) through the post to the local team at their treating hospital or bring it with them if they are returning to hospital for another consultation or treatment. Participants will be randomised to one of the two treatment groups following consent, completion and receipt of baseline questionnaire.

### Randomisation and allocation

Eligible and consenting participants will be randomised to one of the two intervention groups using the telephone Interactive Voice Response (IVR) randomisation application or via the web-based application - both hosted by the Centre for Healthcare Randomised Trials (CHaRT), Health Services Research Unit (HSRU) in Aberdeen, which is a fully registered, Clinical Trials Unit (CTU) with the UK Clinical Research Collaboration (UKCRC).

Randomisation will be minimised by centre, stone size (≤ 10 mm or > 10 mm, as measured by the maximum stone diameter on CTKUB) and location of the stone in either the upper, mid or lower ureter (as defined in the EAU/AUA Guidelines).

### Trial procedures

Participants will complete a total of five questionnaires. They will be asked to complete the EQ-5D-3L, SF-12, pain score (Numerical Rating Scales [NRS]) and use of analgesics at baseline in the hospital when recruited to the trial. Participants will be asked to complete the EQ-5D-3L, pain score and use of analgesics questions (self-completed) at their allocated intervention visit to secondary care immediately before receiving their interventions. At one week post intervention they will be asked to complete the pain score and use of analgesics questions by self-completed questionnaire. At eight weeks and then six months post randomisation, participants will be asked to complete a questionnaire to measure the EQ-5D-3L, SF-12, pain score (NRS) (eight weeks only), use of analgesics, complications, additional interventions received and acceptability of the received procedure. In addition, at six months post randomisation, participants will be asked to complete questions relating to their primary and secondary care use.

Four case report forms (CRFs) will be completed by the research team at the recruiting site. A baseline CRF will be completed at randomisation of the patient. A treatment CRF will be completed following the randomised intervention. The CRFs at eight weeks and six months post randomisation will be completed and entered at site by the centre coordinators at the recruiting centres. They will collect additional interventions received and reasons for those, reasons why they might not have received their allocated intervention, complications and date of stone passage. Additional hospital visits will be recorded on a supplementary CRF.

#### Subject withdrawal

Participants will remain on the trial unless they chose to withdraw consent or if they are unable to continue for a clinical reason. If a participant withdraws consent, participant questionnaires will not be collected; however, permission will be sought for the research team to continue to collect outcome data from their healthcare records (via the CRFs). All other changes in status with the exception of formal withdrawal of consent will mean the participant is still followed up for all study outcomes wherever possible.

### Outcome measures

The study has a primary clinical and a primary economic outcome.

#### Primary outcomes

##### Clinical

Resolution of stone episode defined as no further intervention required to facilitate stone clearance up to six months from randomisation.

##### Economic

Incremental cost per QALYs gained at six months from randomisation. QALYs are based on the responses to the EQ-5D-3L.

#### Secondary outcomes

##### Patient-reported:

- Health state (EQ-5D-3L) at pre intervention, at one week post intervention, at eight weeks and at six months post randomisation.
- Pain intensity at pre intervention, at one week post intervention and at eight weeks post randomisation.
- Functional generic health and wellbeing (SF-12), at eight weeks and at six months post randomisation.
- Analgesic use at pre intervention, at one week post intervention and at eight weeks post randomisation.
- Acceptability of received procedure at eight weeks post randomisation.

##### Clinical:

- Further interventions received up to six months post randomisation.
- Complications up to six months post randomisation.

##### Economic

- NHS primary and secondary care use and costs up to six months.
- Patient costs up to six months.
- Incremental cost per surgical interventions averted.

#### Timing and measures used to assess outcome

Table 1 shows the schedule of outcome assessment and data collection.

**Table 1:** Source and timing of outcome measures

Outcome measures	Source	Timing				
			Intervention		Post randomisation	
		Recruitment	Pre	1 week post	8 weeks	6 months
Additional interventions received	CRF & PQ				✓	✓
Pain (NRS)	PQ	✓	✓	✓	✓	
Health status EQ5D	PQ	✓	✓	✓	✓	✓
Health profile SF-12	PQ	✓			✓	✓
Use of analgesics	PQ	✓	✓	✓	✓	
Complications	CRF				✓	✓
NHS primary and secondary healthcare use	PQ & CRF					✓
Participant costs	PQ					✓

The EQ-5D-3L is used to measure health state today. The pain intensity measure is a NRS [10] using the question ‘Please rate the level of pain that you are experiencing today?’ plus at one week post intervention only ‘Please rate the worst level of pain that you have experienced since your trial treatment?’ The NRS is a segmented numeric scale. The respondent selects a whole number (0–10 integers) that best reflects the intensity of pain. This scale is a horizontal line. The scale is anchored by terms describing pain severity extremes 0 (no pain) to 10 (worst imaginable pain). In addition, participants are asked ‘During the last 7 days have you had pain related to your ureteric stone?’ Yes or No.

The SF-12 measures functional health and wellbeing over the past four weeks summarised in the Physical Component Summary (PCS) and Mental Component Summary (MCS) scores.

Analgesic use is measured with the question ‘How many days out of the last seven have you used pain relief medication?’

The acceptability of the procedure is measured using the question ‘Would you recommend the treatment to a friend?’ In addition, the participant is asked to rate the importance of a list of attributes of treatment: duration of hospital stay; need for further treatment; no complications; pain after treatment; delay in resuming normal daily activities; time off work where 1 = least important and 6 = most important.

### Safety

#### Timing and recording of safety parameters

The TISU trial involves procedures for treating ureteric stone which are well established in clinical practice. Adverse effects may occur during or after any type of surgery.

#### Procedures for recording and reporting adverse events (AEs) and serious adverse events (SAEs)

##### Assessing and recording AEs and SAEs

Non-serious events will not be collected or reported. Planned hospital visits for conditions other than those associated with the ureteric stone will not be collected or reported. Hospital visits (planned or unplanned) associated with further interventions to facilitate ureteric stone clearance will be recorded as an outcome measure but will not be reported as SAEs.

Within TISU, ‘relatedness’ is defined as an event that occurs as a result of a procedure required by the protocol, whether this procedure is the specific intervention under investigation and whether it would have been administered outside the study as normal care.

Any SAEs related to the participants’ ureteric stone treatments that are not further interventions to facilitate stone clearance (e.g. if a participant is admitted to hospital for treatment of infection) will be recorded on the SAE form. In addition, all deaths for any cause (related or otherwise) will be recorded on the SAE form.

The trial office, with the assistance of the CI, will prepare a summary of all serious adverse reactions every six months. These will be distributed to the participating investigators, the Co-Sponsors, the trial steering committee and the DMC.

In addition, all suspected serious adverse reactions will be collated annually and submitted to the REC in accordance with the guidance on annual safety reporting.

The DMC will regularly assess the safety data collected for the trial and will have ability to advise that the trial is temporarily or permanently halted based on safety concerns according to the criteria defined in their charter.

### Sample size

The original sample size calculations reflect that the TISU trial is a non-inferiority design. Published literature [1, 8, 9] suggests the proportion stone free without further intervention up to six months in the ureteroscopy arm will be about 0.75 (P1) and in the ESWL arm about 0.65 (P2). The margin of inferiority deemed acceptable is 0.20 so that P2-P1 > − 0.20. The sample size was estimated using simulations, designed for this purpose, run in Stata. The power of a non-inferiority trial can be considered as the probability that the lower bound of the estimated 95% CI around the difference between trial proportions excludes the margin of non-inferiority. Simulating 1000s of trials of fixed sizes with the parameters P1 and P2 as above indicates that a trial of 450 per arm is required for the lower bound of the estimated 95% CI to exclude − 20% with 90% power. Adjustment for potential 10% drop-out inflates the trial to 1000 in total. A trial of this size would have above 90% power to test superiority on secondary outcomes of an effect size of one-quarter of a standard deviation.

Following poor recruitment and interim data analysis of 267 participants, the sample size was amended from 1000 downwards to 750. The amendment has been ratified by the trial oversight committees and the sponsor and the funder. Recruitment projections showed us that the original sample size of 1000 was unachievable in a realistic timeframe despite measures implemented to improve recruitment. We agreed with the funder an extension of 18 months to reach a revised sample size of 750. Our original sample size of 1000 included a 15% uplift from 850 to perform the primary analysis under a suitably defined per-protocol analysis. A per-protocol analysis, in the special context of a non-inferiority design, is often seen as the more conservative approach than the conventional intention-to-treat (ITT) analysis.

This per-protocol analysis would have excluded any participants that crossed over from their randomly allocated treatment to the other and would have excluded any participant whose stone cleared before their randomly allocated treatment was initiated. However, the view of the HTA Board, which has since been confirmed by the TISU independent Data Monitoring Committee (iDMC), is that the ITT approach should take primacy over this per-protocol approach, to better reflect that the purpose of this pragmatic effectiveness trial is to compare the policy of initiating one or other of these treatment options and not to focus on the actual comparative performance of the treatment options themselves. The per-protocol approach will still be used as a supporting analysis. This is also a rare context in which there is no missing outcome data, since we are sure that we will have identified any further interventions to facilitate stone clearance in the six months postoperatively; hence, we can say with certainty for each individual randomised whether the original operation cleared the stone.

Based on the 267 participants with mature outcome data as of 16 February 2016, the iDMC observed that all the assumptions behind the power calculation remain plausible. Given that we are now committed to the ITT approach rather than the per-protocol approach (and under the original design of assuming the proportion stone free in ESWL arm would be 65% and 75% in the ureteroscopy arm) the achievable sample size of 750 will give 85% power. If we recruit 750, under the assumption of 15% crossover and stone clearance, before initiation of the randomly allocated intervention, then the per-protocol analysis of 638 individuals will still retain ~ 80% power under the same assumptions as above.

### Statistical analysis

Treatment groups will be described at baseline and follow-up using means (with standard deviations), medians (with inter-quartile ranges) and numbers (with percentages) where relevant. Primary and secondary outcomes will be compared using generalised linear models, with adjustment for participant baseline and design covariates, (stone ≤ 10 mm and stone > 10 mm; location in ureter: upper, mid or lower; age; and gender). The measures and timings of outcomes are described in the ‘Outcome measures’ section and Table 1.

Statistical analysis will be per-protocol and ITT (as is recommended for non-inferiority trials) with results displayed as estimates and 95% CIs derived from appropriate generalised linear models. CIs around observed differences will then be compared to the pre-specified non-inferiority margin. Subgroup analyses (appropriately analysed by testing treatment by subgroup interaction) will explore the possible effect modification by type and location of stone and gender; all using stricter levels of statistical significance (*p* < 0.01, 99% CIs).

All analyses will follow a carefully documented Statistical Analysis Plan (SAP). The SAP will be available to both the Trial Steering Committee (TSC) and the iDMC members should they wish to review and comment on the document. A single main analysis will be performed at the end of the trial when all follow-up has been completed.

### Economic evaluation

Economic evaluation will be an integral part of the study. Resource use and costs will be estimated for each participant. The evaluation will consider the costs of the care pathways that patients receive. Resource data collected will include the costs of the interventions, ESWL and ureteroscopy, and simultaneous and consequent use of primary and secondary NHS services (including additional interventions received) by participants. Personal costs, such as purchase of medications, particularly analgesics, time and travel, will also be estimated. The perspective of the study will be societal as it will include both the NHS costs as well as that of the participants.

#### Collection of data

Primary and secondary care resource use will be collected via the CRFs and participant questionnaires. Unit costs will be based on routine sources (e.g. Reference Costs or study specific estimates). QALYs will be based on the responses to the EQ-5D-3L.

#### Participant costs

Participant costs will include self-purchased healthcare such as prescription costs and over-the-counter medications, particularly analgesics.

#### NHS health service resource use

Use of secondary care services following the treatment period will be collected using participant questionnaires and CRFs. Information on outpatient visits, readmissions relating to the use and consequences of the interventions being compared will be recorded. Use of primary care services such as prescription medications, contacts with primary care practitioners, e.g. GPs and practice nurses, will be collected via the ‘health care utilisation questions’ administered at the six-month follow-up.

#### Cost-effectiveness

The cost-effectiveness will be measured in terms of costs of the treatment care pathways and QALYs at six months. QALYs will be estimated by transforming the EQ-5D-3L scores (collected at baseline, eight weeks and six months post randomisation) into utility values using standard algorithms [11]. The results will be presented as point estimates of mean costs, QALYs and incremental cost per QALY of each treatment care pathway. Measures of variance for these outcomes are likely to involve bootstrapping estimates of costs and incremental QALYs. Incremental cost-effectiveness data will be presented in terms of cost-effectiveness acceptability curves (CEACs). Forms of uncertainty, e.g. concerning the unit cost of resources from the different centres, will be addressed using standard deterministic sensitivity analysis. Sensitivity analysis will also be used to explore the impact of statistical imprecision and other forms of uncertainty. Where feasible the results of the sensitivity analyses will also be presented as CEACs.

## Discussion

The TISU trial is a large, multicentre, pragmatic RCT to determine the clinical and cost-effectiveness of a care pathway that starts with ESWL compared to a care pathway that starts with ureteroscopic treatment, for people who have a stone in the ureter that requires an active intervention. Clinical and cost-effectiveness is determined with respect to: (1) resolution of stone episode; (2) incremental cost per QALYs; (3) participant-reported health outcomes; and (4) disease or treatment-related harms up to six months post randomisation.

**Fig. 2:**
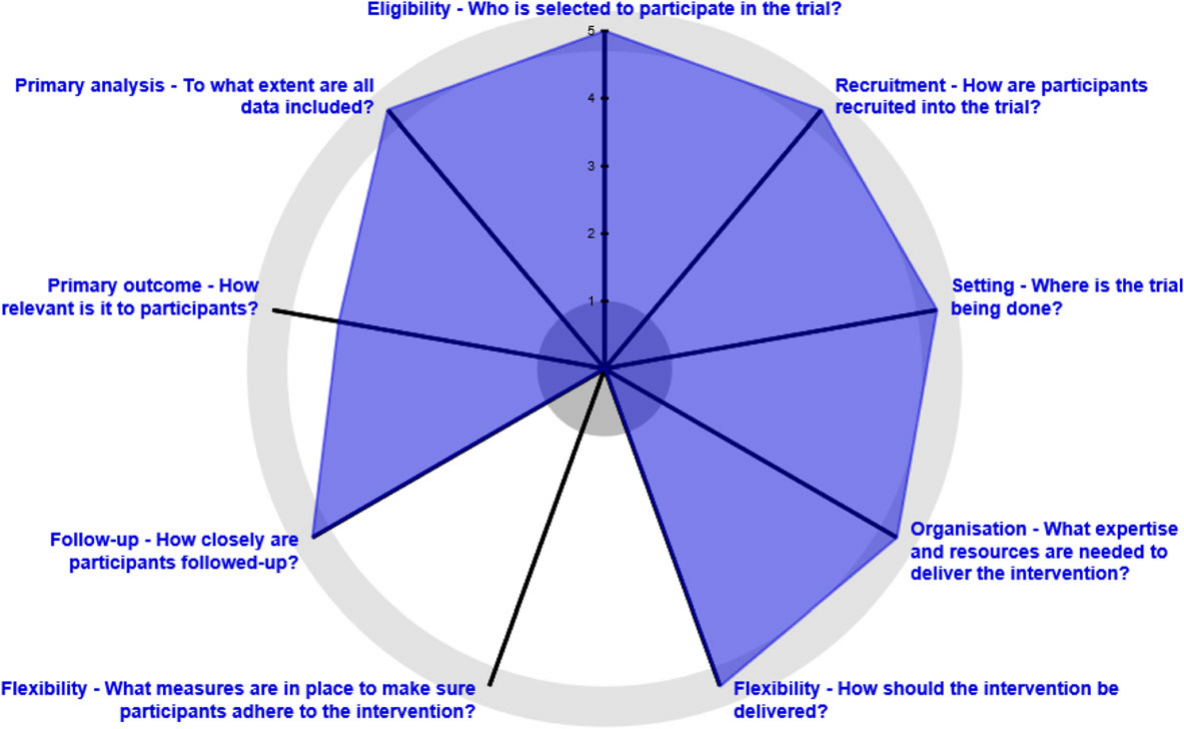
PRECIS-2 wheel for the TISU trial

The trial has been scored using the PRECIS-2 wheel [12] (see Fig. 2: Diagram 1).

It is not possible to blind the TISU trial participants or clinicians to their allocated initial treatments or any further treatments. Lack of blinding may increase threats to the internal validity, such as systematic differences in withdrawal/follow-up, in care provided, in outcome observation, measurement and assessment. In the context of the TISU trial where we are evaluating the effectiveness of a care pathway that starts with concealed random allocation to the initial treatment for a symptomatic condition, we do not think lack of blinding is a major threat to internal validity. The primary outcome is ‘Further interventions to resolve the stone episode up to six months post randomisation’. The outcome is determined via CRF of interventions, collected and collated by research nurses at sites. Standard care guidelines guide treatment decisions and we would not expect clinicians to withhold further interventions judged necessary to facilitate resolution of the stone episode or to withhold treatments for complications over the six months after allocation.

One of the main continuing challenges of the TISU trial is identification and recruitment of participants. The care pathway for ureteric stone patients can be complicated and many patients present to secondary care through Accident and Emergency departments, out of normal hours and are missed by research staff at site. To overcome this issue, many sites are running stone management clinics involving research staff to help identify participants. In other instances, the radiographer at the participating sites is providing researchers with a list of patients that have a single ureteric stone identified by CTKUB. This has helped to ensure that potential patients are not missed.

In addition, feedback from screening logs, qualitative sub-study TISU Qual and discussions with sites have indicated that study recruitment has been affected by patients and clinicians having a preference for one treatment over the other. This is being investigated further to understand the reasons for these preferences and whether measures can be put in place to place them in equipoise when considering the study. The impact of this will be discussed in the full report.

Our previous experience with this patient population has shown that response rates to participant questionnaires tend to be low (49% at 12-week questionnaire follow-up as demonstrated in the SUSPEND trial, August 2015). In anticipation of this, strategies to ensure a better response rate were implemented at the start of TISU. One of these approaches was to provide participants with the choice of how they would prefer to receive questionnaires either by post or via email. The response rate to study questionnaires at six months has always been low and we have observed a slow decrease in the rate. The current rate is 65% completion at six-month time-point. The overwhelming evidence from a recent review of strategies to improve retention in a clinical trial supports the use of monetary incentives in improving response rates [13]. Based on this, we therefore plan to include a monetary incentive (unconditional £10 gift voucher for high street stores) with the participant’s six-month questionnaire to try and improve response rates. Again, the impact of this measure will be discussed in the full trial report.

Other than the problems mentioned there have been no other issues conducting the TISU trial. We have used the recruitment experience on TISU to advise researchers conducting similar trials encouraging them to consider the practicalities of identifying and recruiting patients.

### Trial status

The first participant was recruited in July 2013 and the trial is closed to recruitment and is in follow-up.

## Additional file

Additional file 1: Figure 3 SPIRIT 2013 Checklist: Recommended items to address in a clinical trial protocol and related documents. (DOC 120 kb)

## Abbreviations

CEACs: Cost-effectiveness acceptability curves
CHaRT: Centre for Healthcare and Randomised Trials
CI: Chief investigator
CRF: Case report form
CTKUB: Clinical tomography kidney ureter and bladder
CTU: Clinical Trial Unit
DMC: Data Monitoring Committee
EAU-AUA: European Association of Urology and the American Urological Association
EQ-5D-3L: European Quality of Life – 5 Dimensions - 3 Level
ESWL: Extra-corporeal shock wave lithotripsy
GP: General practitioner
HES: Hospital Episode Statistics
HSRU: Health Services Research Unit
IMP: Investigational medicinal product
ISRCTN: International Standard Randomised Controlled Trial Number
ITT: Intention to treat
IVR: Interactive Voice Response
MET: Medical expulsive therapy
MHRA: Medicines and Healthcare Regulatory Authority
MREC: Research Ethics Committee
NHS: National Health Service
NRS: Numeric rating scale
PI: Principle investigator
QALY: Quality-adjusted life years
RCT: Randomised controlled trial
SAE: Serious adverse event
SAP: Statistical analysis plan
SF-12: Short Form (12) Health Survey
SUSAR: Suspected unexpected serious adverse event
TSC: Trial Steering Committee
UKCRC: UK Clinical Research Collaboration
